# Supplementary material for: Reference Gene Selection for qPCR Analysis in Schima superba under Abiotic Stress
Source: Genes (Basel). 2022 Oct 18;13(10):1887. doi: 10.3390/genes13101887 (PMC9601953; doi:10.3390/genes13101887)
Supplement: Supplementary file 1 [file genes-13-01887-s001.zip › Additional file 3 Figure S2.pdf]

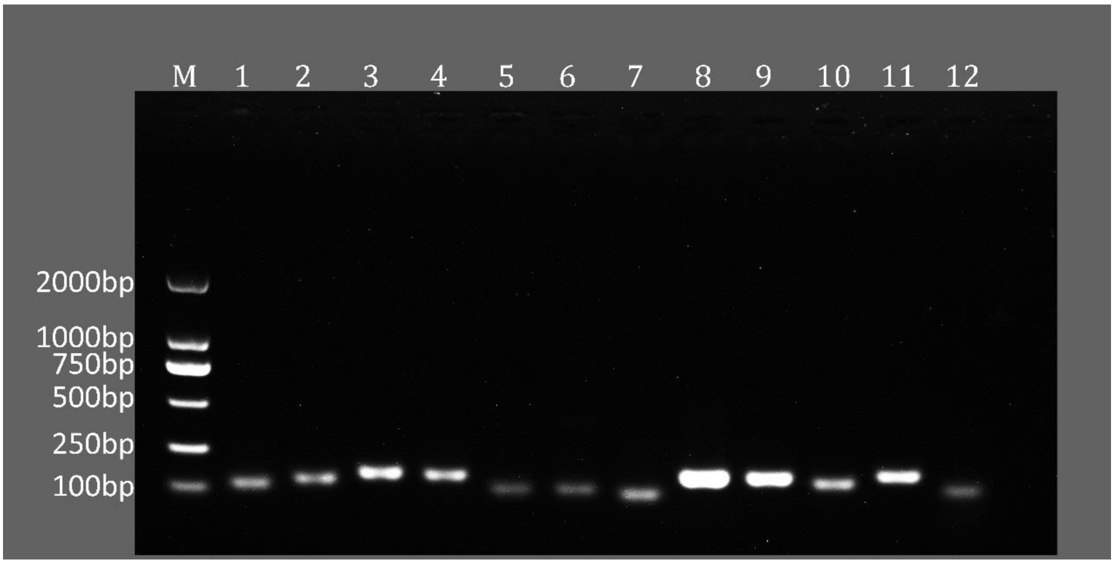

Figure S2. Agarose gel electrophoresis analysis of primer specificity. (1.UBQ, 2. UBC4, 3. GAPDH, 4. TUB, 5. GIIa, 6. UDP, 7. UBC20, 8. AP-2, 9. elF-5a, 10. EF1a, 11.RPL17, 12. Actin)
