# Supplementary figures and images for: Reference Gene Selection for qPCR Analysis in Schima superba under Abiotic Stress
Source: Genes (Basel). 2022 Oct 18;13(10):1887. doi: 10.3390/genes13101887 (PMC9601953; doi:10.3390/genes13101887)

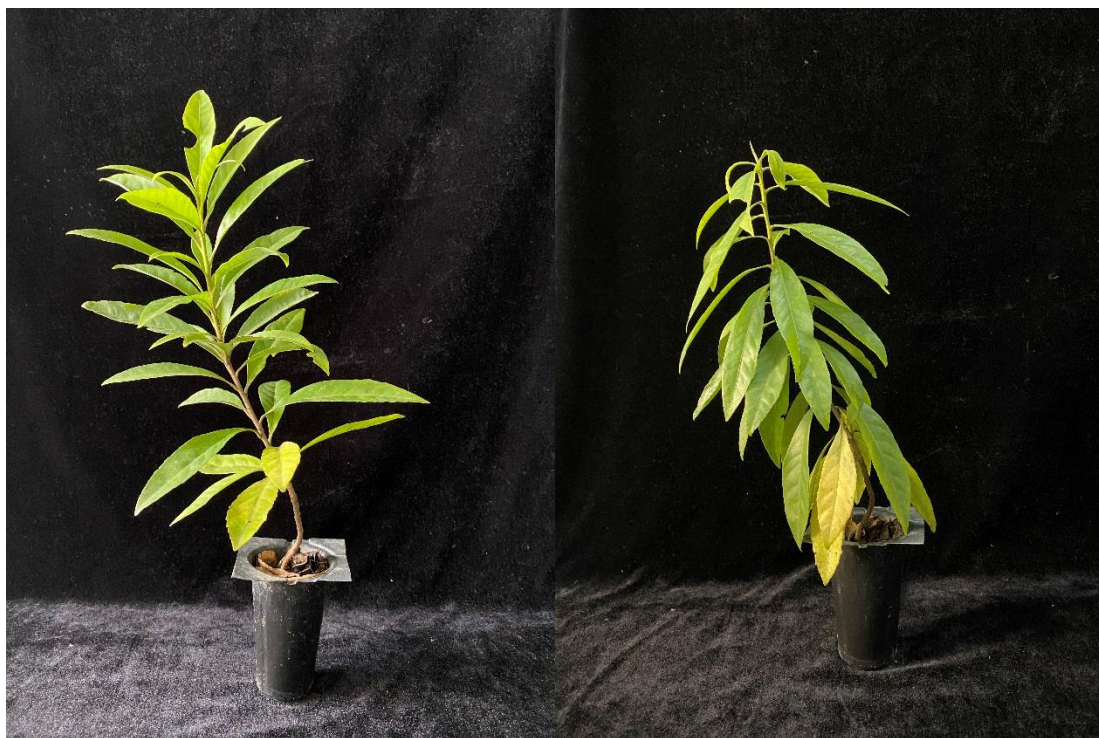

**Figure S1.** Normal appearance and samples under drought stress conditions of *Schima sperba*.

Supplement: Supplementary file 1 [file genes-13-01887-s001.zip › Additional file 1 Figure S1.pdf]

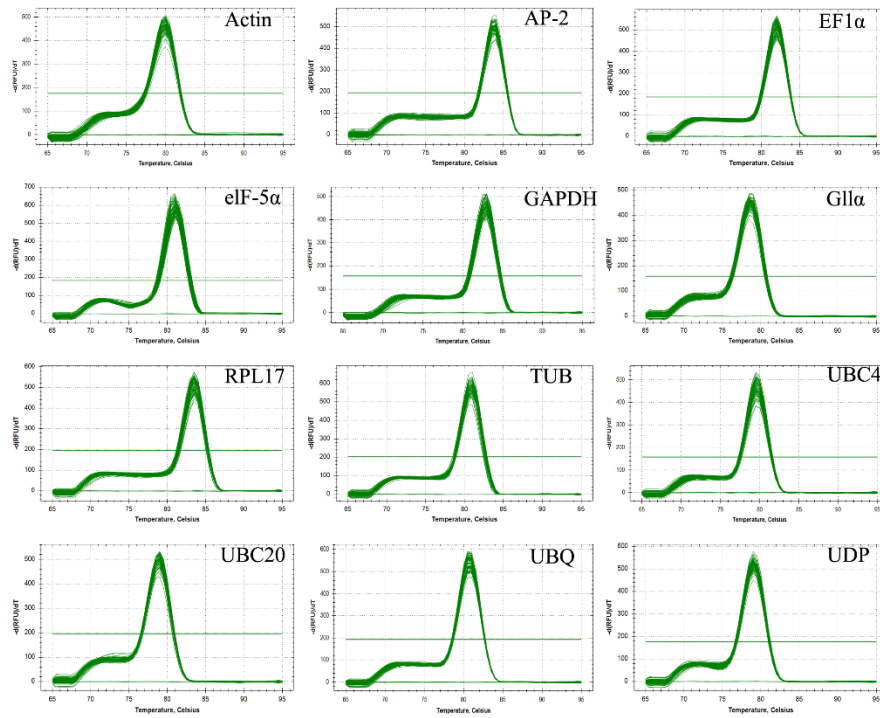

**Figure 3.** Melting curve analysis of twelve reference genes.

Supplement: Supplementary file 1 [file genes-13-01887-s001.zip › Additional file 4 Figure S3.pdf]
